# Tricuspid Valve Endocarditis With Group B Streptococcus After an Elective Abortion: The Need for New Data

**DOI:** 10.1155/IDOG/2006/43253

**Published:** 2006-11-08

**Authors:** Erica E. Palys, John Li, Paula L. Gaut, W. David Hardy

**Affiliations:** Division of Infectious Diseases, Cedars-Sinai Medical Center, 8700 Beverly Boulevard, Becker 220, Los Angeles, CA 90048, USA

## Abstract

*Streptococcus agalactiae*, commonly known as Group B streptococcus (GBS), was originally discovered as a cause of bovine mastitis. GBS colonizes the genital tract of up to 40% of women and has become a major pathogen in neonatal meningitis. GBS endocarditis is thought to be an uncommon manifestation of this infection and carries a higher mortality compared to other streptococcal pathogens. Studies have shown that endocarditis after abortion has an incidence of about one per million. However, this figure was published prior to routine use of echocardiography for diagnosis. The American Heart Association has recently declared transesophageal echocardiography the gold standard for endocarditis diagnosis. This case report illustrates that, given the potentially devastating consequences of endocarditis, there is a need for updated studies to adequately assess the true incidence of this infection. Pending the outcome of these studies, routine GBS screening and prophylactic antibiotics prior to abortion should be recommended.

## INTRODUCTION

*Streptococcus agalactiae*, commonly known as Group B streptococcus (GBS), is a gram-positive coccus and only member of Lancefield group B [1]. Originally discovered as a cause of bovine mastitis [2], it is now a major pathogen in neonatal meningitis [1] and locally invasive as well as serious systemic infections in patients with chronic diseases. GBS endocarditis is thought to be an uncommon manifestation of this infection and is known to carry a higher mortality compared to other streptococcal pathogens [3].

We report a case of a previously healthy young woman, who developed GBS tricuspid valve endocarditis after an elective termination of pregnancy. This case exemplifies the need for strict preoperative antibiotic use for abortions during the second trimester and beyond. It also illuminates our reliance on outdated case reports that have historically stated that the rate of endocarditis after abortion is not high enough to warrant preoperative antibiotics prior to the second trimester. We feel it is time to revisit the issue of whether all women should be screened for GBS infection and if positive receive appropriate antibiotics prior to abortions.

## CASE REPORT

A 22-year-old female with two prior full-term deliveries underwent an elective abortion at 15 weeks gestation at a clinic, 8 weeks prior to admission at our medical center. The procedure was uncomplicated; the patient does not remember receiving antibiotics or being tested for GBS. Her past medical history was noncontributory although she reports a negative HIV test one month prior to admission. She denies using illicit drugs, any injection therapy, or any recent tattoos.

One week after the abortion, she developed low-grade fever and malaise and returned for a repeat cervical dilation and uterine evacuation. Genital cultures grew GBS, *Candida albicans*, and oxacillin-sensitive *Staphylococcus aureus*. She was discharged with 7 days of amoxicillin.

Two weeks later, she presented to another hospital complaining of fever, chills, productive cough, dyspnea, and diffuse chest pain of 5 days duration. She was febrile with enlarged tonsils, no evidence of cardiac abnormalities, rales in bilateral lung bases, and a benign abdomen. A sterile speculum exam revealed yellow-green discharge from the cervix; cultures were sent. She was admitted and treated with ceftriaxone, azithromycin, and metronidazole. CT showed diffuse, patchy infiltrates in the lower lobes with cavitations and no evidence of a pulmonary embolism. Blood cultures grew gram-positive cocci in chains presumed to be *S pneumonia* and nafcillin was added. She left against medical advice and two weeks later presented to our Emergency Department (ED) with worsening fevers, chills, SOB, productive cough, and pleuritic chest pain. In the ED, she was again treated with cefotaxime and azithromycin for presumed community acquired pneumonia and intravenous heparin for possible PE.

On exam she was febrile, hypotensive, tachycardic, and tachypneic. Oxygen saturation was 87% on room air. She had pale conjunctivae and dry mucous membranes. Lung exam revealed diffuse rhonchi and decreased breath sounds on the right. On cardiovascular exam, her neck showed cannon *v* waves along with a 3/6 holosystolic murmur best heard at the lower left sternal border. Abdominal and extremity exams were benign. There were no signs of Osler's nodes or Janeway lesions.

Her CBC revealed a WBC of 19.3, hemoglobin of 9.6, and platelets of 81 000. D-dimer was 4540 ng/mL; beta hCG was negative. Chest radiograph showed bilateral, fluffy, lower lobe infiltrates with peripheral cavitations. Ultrasound of the lower extremities for deep vein thromboses was negative. EKG revealed sinus tachycardia and right ventricular conduction delay (RSR'). CT of the chest, abdomen, and pelvis revealed large, bilateral, peripheral lower lobe cavitary lesions.

**Figure 1:**
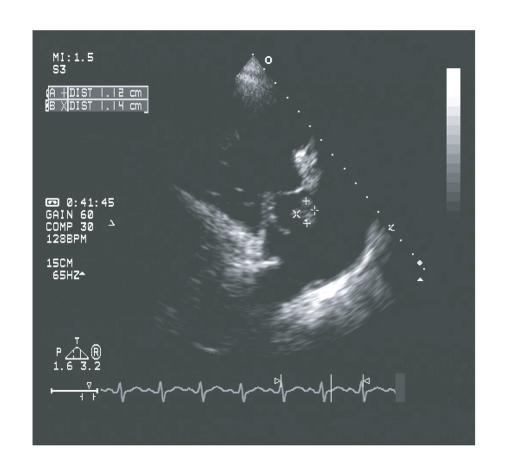
Tricuspid valve vegetation measuring 1.14 cm × 1.12 cm in this view.

She was admitted to the ICU and treated with penicillin G, gentamicin, and clindamycin. Transthoracic echocardiogram showed estimated pulmonary artery systolic pressures of 55 mmHg, right atrial and right ventricular dilation, and large tricuspid valve vegetation with severe tricuspid regurgitation (Figure 1). Left ventricular ejection fraction was 56%.

Blood cultures were reported positive in 12 hours with GBS, sensitive to penicillin (MIC 0.12) and cefotaxime (MIC 0.12). Clindamycin was stopped.

Throughout her stay, the patient was persistently febrile. Repeat CT of the chest, 2 weeks after admission, showed improving pulmonary infiltrates; multiple, bilateral cavitary lesions increased in size; cardiomegaly with a very large right atrium and suprahepatic inferior vena cava, along with a severely enlarged liver suggesting right heart failure.

She was treated with 3 weeks of gentamicin and discharged on continued intravenous penicillin G for a total of 6 weeks of therapy. Although her echocardiograms and CT scans showed evidence of right heart failure, she had no clinical evidence of this; it was decided to monitor the patient closely and forgo surgery. The patient missed two followup appointments after her discharge.

She appeared in the ED a few months later complaining of chest pain and dyspnea on exertion. CT scan of chest showed left descending pulmonary artery aneurysm without clot or dissection but clear evidence of pulmonary hypertension. Echocardiogram in the ED revealed normal LV motion and ejection fraction, complete resolution of the vegetation, pulmonary artery peak systolic pressure of 41 mmHg, a thickened tricuspid valve with severe regurgitation, and septal flattening consistent with right ventricular volume overload. The cardiologist evaluated the patient and recommended her to followup for a possible tricuspid valve replacement. However, the patient was again lost to followup.

## DISCUSSION

Studies have reported rates of GBS colonization in pregnant women of 4.6% to 40.6% [1, 4, 5]. Fifty percent of colonized mothers transmit GBS to their newborns [1]. For this reason penicillin is routinely recommended during delivery for women colonized with GBS. The incidence of maternal complications from GBS colonization is less well described. In general it has been reported to cause benign infections such as urinary tract infections. Previously reported serious maternal complications due to GBS include chorioamnionitis [6] and endometritis, which occur more after cesarean sections than vaginal deliveries [6, 7].

According to a 1973 often-cited article, the incidence of GBS endocarditis following obstetric or gynecological procedures is low, ranging from 0.03 to 0.14 per 1 000 deliveries [5]. Seaworth and Durack's 1986 article provides us with the most up to date estimation of the incidence of infective endocarditis after abortion: about one per million [4]. The pathogenesis of GBS endocarditis is presumed to be bacteremia [8] following manipulation of the genital tract colonized with GBS [9]. In one small study of only 20 patients, bacteremia following routine dilation and curettage occurred in 5% (1 out of 20 patients); the bacterium isolated was *Lactobacillus* [10]. There is a paucity of current studies examining this topic. In the nearly twenty years since Seaworth and Durack's 1986 article, transesophageal echocardiography (TEE) has proven itself to be superior to transthoracic echocardiography (TTE). One of the early studies examining TEE versus TTE was in 1991. It was felt that TEE represented “a significant advance over transthoracic echocardiography,” as TEE had twice the sensitivity of TTE [11]. The 2005 American Heart Association Guidelines on endocarditis state that “[TEE] is the preferred imaging technique for the diagnosis … of infective endocarditis (IE) in adults with either high risk for IE or moderate-to-high clinical suspicion of IE of in patients in whom imaging by [TTE] is difficult” [12]. There is a great chance that Seaworth and Durack—relying upon what would today be considered incomplete, if not outdated, standards—missed cases of endocarditis that could today be diagnosed with TEE. It is also important to note that their data looked at cases from 1940 through 1983 and that echocardiography was not in their diagnostic criteria. They also relied upon published case reports in their estimation of the incidence of endocarditis after abortion being about 1 in 1 million. Because the women undergoing abortions are usually younger and have a high potential to be lost to follow up, this is an important issue to revisit.

Mortality from endocarditis still ranges from 20% to 25%, down from the 35% to 40% reported from the 1960s. The decrease is likely due to earlier detection and improved medical and surgical therapies. However, the numerous potential serious complications of infective endocarditis serve as an important reminder of the significant morbidity associated with this infection. Septic emboli cause the majority of these complications, including but not limited to septic pulmonary emboli, cerebral vascular accidents, renal failure, and myocardial infarction [13]. Other complications include congestive heart failure, arrhythmias, abscess formation—both in the heart and as a consequence of septic emboli elsewhere, osteomyelitis, pneumonia, and meningitis [3, 14].

## FURTHER QUESTIONS

Given the morbidity and mortality related to endocarditis and the relatively easy and cheap preoperative regimen, should all women be screened for GBS and, if positive, given antibiotics prior to all elective abortions? Certainly, all women at risk—those with prosthetic heart valves, known MVP, or other risk factors—should be tested for GBS and given endocarditis prophylaxis [12]. However, what to do with women colonized with GBS and undergoing abortions (especially first trimester) has not been discussed since the advent of new technology. Current treatment guidelines are based upon old literature that must be updated. Information regarding rates of bacteremia after abortion, rates of endocarditis after women colonized with GBS undergo abortion, and better morbidity and mortality data must be sought before the current guidelines can be safely relied upon. Prospective studies are warranted in order to have a more accurate estimate of the rate of endocarditis after abortion, utilizing the new guidelines of endocarditis diagnosis.
